# A phase 1b/2, open-label, dose-escalation, and dose-confirmation study of eribulin mesilate in combination with capecitabine

**DOI:** 10.1038/s41416-018-0366-5

**Published:** 2019-02-20

**Authors:** Chris Twelves, Alan Anthoney, Claudio I. Savulsky, Matthew Guo, Larisa Reyderman, Nicola Cresti, Vladimir Semiglazov, Constanta Timcheva, Ishtiaq Zubairi, Rosemary Morrison, Ruth Plummer, T. R. Jeffry Evans

**Affiliations:** 1 Leeds Institute of Cancer and Pathology, University of Leeds and Leeds Teaching Hospitals Trust, Leeds, UK; 2 Clinical Development Oncology, Oncology Production Creation Unit, Eisai Ltd, Hatfield, UK; 3 Biostatistics, Oncology PCU, Eisai Inc, Woodcliff Lake, NJ USA; 4 Clinical Pharmacology and Translational Medicine, Oncology, Eisai Inc, Woodcliff Lake, NJ USA; 5 Northern Centre for Cancer Care, The Newcastle upon Tyne Hospitals NHS Foundation Trust, Newcastle upon Tyne, UK; 6 Department of Tumors of Reproductive System and Breast Cancer, NN Petrov Research Institute of Oncology, St Petersburg, Russia; 7 Medical Oncology Clinic, Multiprofile Hospital for Active Treatment “Nadezhda” Sofia, Sofia, Bulgaria; 8 Department of Medical Oncology, Beatson West of Scotland Cancer Centre, Glasgow, UK; 9 Clinical Research Unit, Beatson West of Scotland Cancer Centre, Glasgow, UK; 10 Institute of Cancer Sciences, University of Glasgow, Glasgow, UK

**Keywords:** Chemotherapy, Breast cancer

## Abstract

**Background:**

Capecitabine and eribulin are widely used as single agents in metastatic breast cancer (MBC) and have nonoverlapping toxicities.

**Methods:**

In phase 1b (dose escalation), patients with advanced, treatment-refractory, solid tumours received eribulin mesilate intravenously in 21-day cycles according to schedule 1 (day 1) or schedule 2 (days 1, 8) with twice-daily oral capecitabine (1000 mg/m^2^ days 1–14). In phase 2 (dose confirmation), women with advanced/MBC and ≤3 prior chemotherapies received eribulin mesilate at the maximum tolerated dose (MTD) per the preferred schedule plus capecitabine. Primary objectives were MTD and dose-limiting toxicities (DLTs; phase 1b) and objective response rate (ORR; phase 2). Secondary objectives included progression-free survival (PFS), safety, and pharmacokinetics.

**Results:**

DLTs occurred in 4/19 patients (schedule 1) and 2/15 patients (schedule 2). Eribulin pharmacokinetics were dose proportional, irrespective of schedule or capecitabine coadministration. The MTD of eribulin was 1.6 mg/m^2^ day 1 for schedule 1 and 1.4 mg/m^2^ days 1 and 8 for schedule 2. ORR in phase 2 (eribulin 1.4 mg/m^2^ days 1, 8 plus capecitabine) was 43% and median PFS 7.2 months. The most common treatment-related adverse events were neutropenia, leukopenia, alopecia, nausea, and lethargy.

**Conclusions:**

The combination of capecitabine and eribulin showed promising efficacy with manageable tolerability in patients with MBC.

## Introduction

The treatment of advanced or metastatic breast cancer (MBC) remains palliative, with little available evidence to recommend a specific sequence of therapies.^1^ Both American Society of Clinical Oncology and European Society for Medical Oncology guidelines indicate sequential single-agent therapy as a preferred choice for the treatment of MBC in most circumstances.^1,2^ However, combination therapy often achieves higher response rates and longer progression-free survival (PFS) than monotherapy, albeit frequently with increased toxicity and little evidence for significant prolongation of overall survival (OS).^3^ Therefore, there remains a need for an effective and well-tolerated combination therapy for patients with MBC.

Eribulin mesilate (eribulin) is a novel microtubule dynamics inhibitor with mechanisms of action distinct from that of other conventional tubulin-targeting agents.^4–6^ Eribulin also causes vascular remodelling, which may improve tumour perfusion, leading to increased intratumoural drug penetration and potentially enhanced efficacy.^7,8^ Eribulin monotherapy prolongs survival in comparison to treatment of physician’s choice in women with previously treated MBC.^9^ In a randomised phase 3 trial of eribulin vs capecitabine in patients with MBC, eribulin did not significantly improve OS.^10^ However, subgroup analysis from this study showed OS benefit in various subgroups, including patients with human epidermal growth factor receptor 2 (HER2)-negative MBC and triple-negative MBC.^11^ Eribulin is approved for the treatment of patients with MBC whose disease has progressed after at least 1 (European Union) or 2 (United States) prior chemotherapies for advanced/MBC, including an anthracycline and a taxane, given at any stage of the disease.

Capecitabine is a fluoropyrimidine carbamate prodrug of 5’-fluorouracil (5-FU), commonly utilised as a single agent for the treatment of patients with MBC and also in combination with other agents, including docetaxel.^12,13^ Eribulin and capecitabine represent, therefore, two of the most widely used therapies in patients with MBC previously treated with an anthracycline and a taxane. Preclinical evidence in xenograft models suggests at least additive efficacy when eribulin and capecitabine are combined.^14^ Moreover, prior treatment with eribulin enhanced the antitumour activity of capecitabine in the MDA-MB-231 breast cancer xenograft model.^7^ Importantly, the most clinically relevant adverse events (AEs) associated with eribulin (neutropenia, asthenia/fatigue, and peripheral neuropathy)^9,10^ are distinct from those most often seen with capecitabine (diarrhoea and hand–foot syndrome),^10,15^ further supporting the development of this combination.

We conducted a phase 1b/2 study of eribulin in combination with capecitabine. The primary objective of phase 1b was to determine the maximum tolerated dose (MTD) of the combination when administered in two schedules in patients with advanced, refractory cancer. The primary objective of phase 2, the dose-confirmation component, was to determine the objective response rate (ORR) of the combination in patients with MBC. Secondary objectives included safety, time to response, duration of response, duration of stable disease, disease control rate (DCR), clinical benefit rate (CBR), PFS, and pharmacokinetic/pharmacodynamic analyses evaluating a correlation of eribulin plasma concentration with changes in cardiac repolarisation, as measured by change in Fridericia’s formula QT correction (QTcF) from baseline.

## Methods

### Patients

#### Dose-escalation cohorts (phase 1b)

Key eligibility criteria included patients aged ≥18 years with histologically or cytologically confirmed advanced or metastatic cancer resistant or refractory to approved therapies. Additional eligibility criteria are summarised in Supplementary Table S1.

#### Dose-confirmation cohort (phase 2)

Key eligibility criteria included females aged ≥18 years with histologically or cytologically confirmed advanced or MBC; ≤3 prior lines of chemotherapy (sequential [neo-] adjuvant treatments counting as 1 regimen), including an anthracycline (if appropriate) and a taxane; ≥1 measurable lesion according to Response Evaluation Criteria In Solid Tumors (RECIST) version 1.1^16^; adequate haematologic, liver, and renal function; life expectancy of >3 months; and Eastern Cooperative Oncology Group performance status of 0 or 1. Additional eligibility criteria are summarised in Supplementary Table S1. Prior capecitabine treatment was not allowed. Patients with pre-existing neuropathy of grade >2 were ineligible for both phases of the study.

### Study design

This was a phase 1b/2, multicentre, open-label, dose-escalation, and dose-confirmation study (ClinicalTrials.gov, NCT01323530). Phase 1b of the study was conducted in the United Kingdom at 3 sites with the addition of sites in Bulgaria (4) and Russia (6) for phase 2. The phase 1b dose-escalation part had a “3+3” design. Sequential cohorts of 3–6 patients received eribulin mesilate (2–5-min intravenous administration) according to schedule 1 (1.2, 1.6, or 2.0 mg/m^2^ [equivalent to eribulin 1.05, 1.40, and 1.75 mg/m^2^, respectively, expressed as free base] on day 1 of a 21-day cycle) or schedule 2 (0.7, 1.1, or 1.4 mg/m^2^ [equivalent to eribulin 0.62, 0.97, and 1.23 mg/m^2^, respectively, expressed as free base] on days 1 and 8 of a 21-day cycle), in combination with twice-daily oral capecitabine (1000 mg/m^2^ on days 1–14 of a 21-day cycle).

Treatment-emergent AEs (TEAEs) were defined as AEs that were new or had re-emerged and worsened in severity during treatment or up to 30 days following the last dose of study treatment. Toxicities were graded according to the National Cancer Institute’s Common Terminology Criteria for Adverse Events version 3.0. The MTD was defined as the highest dose at which no more than 1 out of 6 patients experienced a dose-limiting toxicity (DLT), determined in phase 1b. DLTs were defined as toxicities considered to be treatment related by the investigator, including grade 3 or 4 neutropenia complicated by fever (≥38.5 °C), or grade 4 neutropenia lasting ≥7 days; grade 3 thrombocytopenia complicated by bleeding and/or requiring a blood transfusion, or grade 4 thrombocytopenia; and ≥grade 3 non-haematologic toxicities (excluding grade 3 nausea, grade 3/4 vomiting, or diarrhoea in patients not having received antiemetic and/or antidiarrhoeal medication), delayed recovery from treatment-related toxicity resulting in a dose delay ≥14 days, and failure to administer at least 75% of the planned study drugs during cycle 1 as a result of a grade ≥2 treatment-related toxicity that constituted an increase of at least 2 grades from baseline (i.e. failure to receive at least 14 doses of capecitabine in schedule 1 or failure to receive eribulin mesilate on day 8 in schedule 2). The criteria for dose interruptions and dose modifications of both agents are included in Supplementary Table S2.

In phase 2, patients received the preferred schedule of eribulin selected based on tolerability and delivered dose intensity in phase 1b. In both study phases, treatment continued until disease progression, unacceptable toxicity, or withdrawal of consent. Following dose reduction, dose re-escalation was not permitted. Patients with metastatic bone disease being treated with bisphosphonates could continue to receive bisphosphonate therapy and the initiation of bisphosphonates after starting study treatment was permitted upon discussion with the sponsor.

This study was conducted in accordance with national and local Good Clinical Practice guidelines and the Declaration of Helsinki and was approved by the relevant Institutional Review Board ethics committees. All patients provided written informed consent prior to participating in any study-specific procedures.

### Study assessments

Tumour response per RECIST version 1.1 was assessed by the investigator (Supplementary Table S3). The ORR was defined as the proportion of patients with a confirmed complete response (CR) or partial response (PR). Stable disease (SD) was defined as SD lasting ≥5 weeks; duration of SD was measured from time of first dose until progression in patients whose best response was SD. DCR comprised CR, PR, and SD. CBR comprised CR, PR, and SD ≥6 months.

### Pharmacokinetic assessments

Blood samples were taken during cycles 1 and 2 on day 1 (pre-dose, start of infusion, and 0.25, 0.5, 1, 2, 3, 4, 6, and 8 h after eribulin administration), on days 2 or 3 and days 4 to 6, then on day 8 (schedule 1: pre-dose, 0.5, 1, 2, 3, 4, and 6 h after capecitabine administration; schedule 2: pre-dose). Plasma concentrations of eribulin, capecitabine, and the metabolites of capecitabine (5-FU, 5’-deoxy-5-fluorouridine, 5’-deoxy-5-fluorocytidine, and α-fluoro-β-alanine) were measured by fully validated methods of liquid chromatography–tandem mass spectrometry.^17^ Pharmacokinetic parameters, including clearance (CL; for eribulin), area under the concentration–time curve from time zero to infinity (AUC_0–∞_), area under the concentration–time curve from time zero to the time of last measurable concentration (AUC_0–*t*_), maximum plasma concentration (*C*_max_), time to *C*_max_ (*t*_max_), and terminal half-life (*t*_1/2_), were calculated using noncompartmental analyses as appropriate.

To assess potential drug–drug interactions between eribulin and capecitabine, the eribulin pharmacokinetic parameters *C*_max_, CL, and AUC_0–∞_ were compared to historical data for eribulin administered alone. These parameters were also compared between cycle 1 and cycle 2 to assess for potential indirect/time-dependent effects of capecitabine on eribulin pharmacokinetics. The pharmacokinetics of capecitabine were compared between groups from schedules 1 and 2 for which, on cycle 1 day 8, an assessment of a potential for a direct PK interaction could have been made. Capecitabine pharmacokinetics following single and multiple doses from the dose-escalation and intensive pharmacokinetic sampling cohorts were compared with published pharmacokinetic data on capecitabine.

For cardiac repolarisation analyses, individual electrocardiograms (ECGs) were obtained in triplicate from continuous 12-lead Holter recordings at timepoints coinciding with pharmacokinetic sample collections. Cardiac repolarisation was measured as the duration of the QT interval (time from QRS complex to end of T wave). QT interval was corrected for heart rate by QTcF. Triplicate QTcF values for each patient were averaged by timepoint to provide a single data point per observation. The relationship between time-matched drug concentration and change in QTcF from baseline (ΔQTcF) was analysed using linear regression. Because capecitabine was co-administered with eribulin, ΔQTcF could not be attributed to a single drug or analyte; therefore, the explored concentration–QTcF relationships were considered to be descriptive.

### Statistical analyses

Of the estimated 76 patients to be treated, approximately 40 women with advanced breast cancer or MBC were to be enrolled in the phase 2 study to enable efficacy and safety evaluations. If the ORR was 30%, a sample size of 40 patients was required to provide 2-sided 95% confidence intervals (CIs) with a lower limit of 16%.

Safety analyses were based on the safety population, which comprised all patients who received the study drug and had at least one post-dose safety assessment; efficacy analyses were based on the full analysis set that comprised all enrolled patients who had received at least 1 dose of the study drug. The phase 2 secondary efficacy endpoints of time to response (defined as the time from the first dose until first documented evidence of CR or PR), duration of response (defined as the time from first documented evidence of CR or PR until the first documented sign of disease progression or death due to any cause), duration of SD, and PFS (defined as the time from the date of first dose until disease progression or death due to any cause) were summarised using Kaplan–Meier plots. For DCR and CBR, the exact Clopper–Pearson 2-sided 95% CIs were estimated.

## Results

### Dose-escalation cohort (phase 1b)

**Fig. 1:**
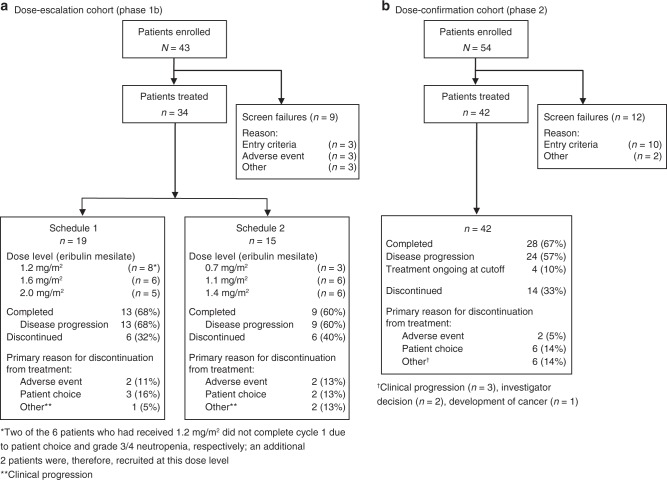
Patient disposition and primary reason for discontinuation from study treatment (combining treatment phase and extension phase)

Thirty-four patients were treated in the phase 1b study (schedule 1, *n* = 19; schedule 2, *n* = 15; Fig. 1). Baseline demographics and disease characteristics for patients in phase 1b are summarized in Supplementary Table S4. With schedule 1, 1 patient had a DLT at the 1.2-mg/m^2^ dose level (febrile neutropenia, grade 3); 1 had a DLT at the 1.6-mg/m^2^ dose level (neutropenia, grade 4); and 2 had a DLT at the 2.0-mg/m^2^ dose level (fatigue, grade 3; lethargy, grade 3). With schedule 2, 1 patient had a DLT at the 1.1-mg/m^2^ dose level (neutropenic sepsis, grade 4), and another patient had a DLT at the 1.4-mg/m^2^ dose level (neutropenia, grade 3). For schedules 1 and 2, the MTDs for eribulin mesilate (in combination with capecitabine 1000 mg/m^2^ twice daily) were 1.6 mg/m^2^ on day 1 and 1.4 mg/m^2^ on days 1 and 8, respectively. The planned dose intensity for schedule 1 was 0.076 mg/m^2^/day and for schedule 2 was 0.13 mg/m^2^/day. Because schedule 2 could provide a better dose intensity than even the highest dose level in schedule 1, only the MTD determined during schedule 2 was selected for further exploration. A summary of TEAEs is provided in Table 1; no treatment-related deaths occurred.

**Table 1:** Extent of exposure and the incidence of grade 3 and 4 treatment-related treatment-emergent adverse events reported for each schedule (worst grade of all cycles) in the dose-escalation cohort (phase 1b, safety analysis set)

Characteristic	Schedule 1	Schedule 2
	(*n* = 19)	(*n* = 15)
Number of complete treatment cycles (%)		
0–2	13 (68)	4 (27)
3–4	2 (11)	3 (20)
5–8	1 (5)	6 (40)
>8	2 (11)	2 (13)
Dose modifications of eribulin mesilate (%)		
Dose reduction	2 (11)^a^	4 (27)^b^
Dose omission	0	4 (27)^b^
Dose delay	4 (21)	9 (60)^c^
Eribulin dose intensity at MTD (mg/m^2^/day) per patient, mean (SD)	0.37 (0.6)^d^	0.16 (0.1)^d^
Grade 3/4 treatment-related TEAE (%)		
Neutropenia	4 (21)	7 (47)
Lethargy	1 (5)	2 (13)
Fatigue	1 (5)	1 (7)
Febrile neutropenia	1 (5)	0
Neutropenic sepsis	0	1 (7)
Pyrexia	0	1 (7)
Palmar-plantar erythrodysaesthesia	0	1 (7)
Pulmonary embolism	0	1 (7)
QTc prolongation	0	1 (7)
Leukopenia	0	1 (7)
TEAE(s) leading to discontinuation of study drug		
Febrile neutropenia	1 (5)^e^	—
Neutropenia	1 (5)^e^	1 (7)^f^
Abnormal ECG	—	1 (7)^g^

Data presented as *n* (%) unless stated otherwise. Safety analysis set: all patients who received both study drugs and had at least 1 post-dose safety assessment. Treatment-related TEAEs include TEAEs that were considered by the investigator to be possibly or probably related to study drug or TEAEs with missing causality

*ECG* electrocardiogram, *MTD* maximum tolerated dose, *QTc* QT interval corrected for heart rate, *SD* standard deviation, *TEAE* treatment-emergent adverse event

^a^In the eribulin mesilate 2.0-mg/m^2^ cohort

^b^2 patients each in the eribulin mesilate 1.1- and 1.4-mg/m^2^ cohorts

^c^1, 3, and 5 patient(s) each in the eribulin mesilate 0.7-, 1.1-, and 1.4-mg/m^2^ cohorts, respectively

^d^6 patients each in the eribulin mesilate 1.6-mg/m^2^ (schedule 1) and 1.4-mg/m^2^ (schedule 2) cohorts

^e^Grade 3 TEAE in the eribulin mesilate 1.2-mg/m^2^ cohort

^f^Grade 4 TEAE in the eribulin mesilate 1.4-mg/m^2^ cohort

^g^Grade 1 TEAE in the eribulin mesilate 1.4-mg/m^2^ cohort

#### Pharmacokinetics and pharmacodynamics

**Fig. 2:**
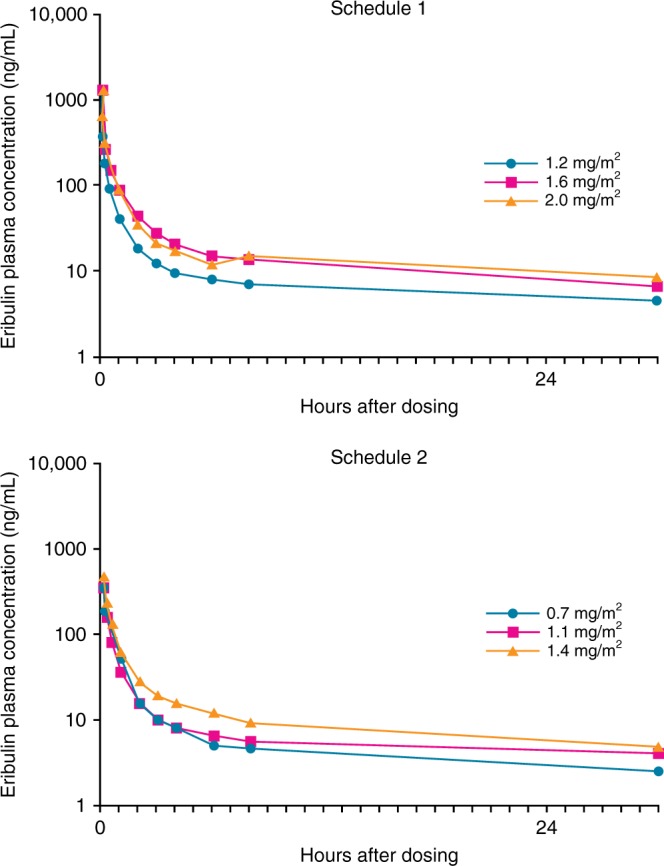
Mean eribulin plasma concentration–time profiles (phase 1b, schedule 1, day 1; schedule 2, day 1)

Eribulin exposure was dose proportional from 0.7 to 2.0 mg/m^2^ on day 1 of cycles 1 and 2 (Fig. 2, Supplementary Table S5). Following single-dose administration of eribulin on cycle 1, day 1 only, mean (±SD [standard deviation]) values for AUC_0–∞_ in schedule 1 ranged from 606 (±288) ng·h/mL in the 1.2 mg/m^2^ group to 1480 (±979) ng·h/mL in the 1.6 mg/m^2^ group. In schedule 2, mean (±SD) values for AUC_0–∞_ ranged from 422 (±48) ng·h/mL in the 0.7 mg/m^2^ group to 785 (±258) in the 1.4 mg/m^2^ group (Supplementary Table S5). Mean (±SD) AUC_0–∞_ in schedule 1 for eribulin in cycle 2, day 1, ranged from 578 (±449) ng·h/mL in the 1.2 mg/m^2^ group to 1530 (±1120) ng·h/mL in the 1.6 mg/m^2^ group. In schedule 2, mean (±SD) values for AUC_0–∞_ ranged from 425 (±73) ng·h/mL in the 1.1 mg/m^2^ group to 686 (±110) in the 1.4 mg/m^2^ group (Supplementary Table S5). The pharmacokinetics of eribulin were independent of schedule or coadministration of capecitabine.

The pharmacokinetic-parameter estimates for capecitabine and its metabolites were comparable between cycle 1 and cycle 2. Coadministration with eribulin had no effect on the pharmacokinetics of capecitabine or its metabolites (Supplementary Fig. S1 and Table S6). Furthermore, comparable exposure was observed across eribulin dose groups and cycles (Supplementary Fig. S1). No correlation was seen between eribulin concentration (day 1 or day 8) and change in QTcF interval in the dose-escalation phase 1b (Supplementary Fig. S2).

### Dose-confirmation cohort (phase 2)

Of the 42 patients treated in the phase 2 study (Fig. 1), 33 (79%) had HER2-negative disease and 16 (38%) had triple-negative disease (Table 2). All patients had received prior cancer therapy, with 34 (81%) patients having received ≥2 regimens and all patients having received prior anthracycline therapy (Table 2). Thirty-three (79%) patients had received prior chemotherapy for advanced disease. Twenty-three (55%) patients entered an extension phase (starting at week 18) and received treatment for as long as clinically appropriate.

**Table 2:** Baseline patient and disease characteristics for the dose-confirmation cohort (phase 2)

Characteristic	Phase 2 (*n* = 42)
Median age, years (range)	52.5 (32–74)
Gender, *n* (%)	
Female	42 (100)
Race, *n* (%)	
White	41 (98)
Black	1 (2)
ECOG PS, *n* (%)	
0	18 (43)
1	24 (57)
Metastases at study entry, *n* (%)	
Liver	20 (48)
Lung	24 (57)
HER2 status, *n* (%)	
+	4 (10)
−	33 (79)
Not done or unknown	5 (12)
Triple-negative disease^a^, *n* (%)	16 (38)
Prior chemotherapy regimens, *n* (%)	
1	8 (19)
2	16 (38)
3	12 (29)
>3	6 (14.3)
Prior anthracycline therapy	42 (100)
Type of prior chemotherapy, *n* (%)	
Adjuvant	28 (66.7)
Neoadjuvant	10 (23.8)
Advanced	33 (78.6)
Median time from first diagnosis to study entry, months (range)	29.0 (3–181)
Median age at diagnosis, years (range)	49.0 (30–71)
Median time since last disease progression, months (range)	1.6 (1–10)
Median duration of last therapy, months (range)	2.8 (0–106)

Best response from last therapy was not collected for this cohort

*ECOG PS* Eastern Cooperative Oncology Group performance status, *ER* oestrogen receptor, *FISH* fluorescence in situ hybridisation, *HER2* human epidermal growth factor receptor 2, *PR* progesterone receptor

^a^Triple-negative status defined as: Yes (HER2 0/1+ or 2+ with FISH negative, ER negative, and PR negative); and No (other cases)

**Fig. 3:**
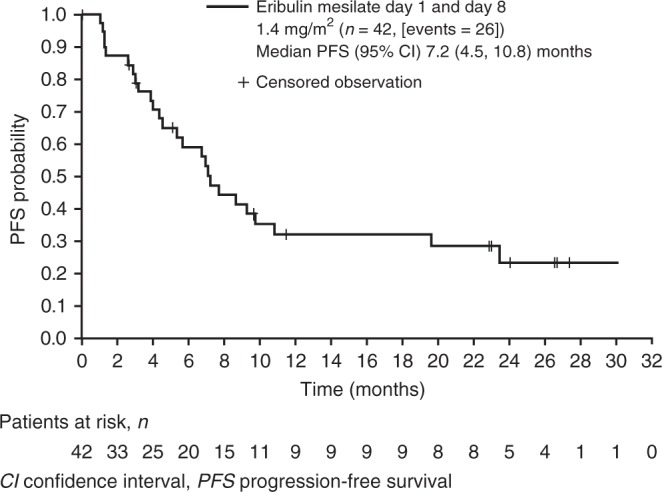
Kaplan–Meier estimates of progression-free survival in the dose-confirmation cohort (phase 2) for all patients

The ORR was 18 out of 42 (43%; 95% CI 27.7, 59.0), with 1 CR and 17 PRs; responses were seen in patients with HER2-negative/hormone receptor+ disease (9/17 patients), triple-negative disease (6/16), HER2-positive disease (1/4), and HER2 unknown (2/5) (Supplementary Table S7). Median time to response was 1.5 months, and median duration of response was 8.6 months. SD was observed in 16 (38%) patients, with a median duration of 5.3 months; 3 (7%) patients had progressive disease (PD) as their best response. The DCR was 81%, and the CBR was 57% (Supplementary Table S7). Median PFS was 7.2 months overall (Fig. 3).

In phase 2, the median number of treatment cycles was 8 (Supplementary Table S8). Overall, 39 (93%) patients experienced ≥1 TEAE in this phase of the study, 38 (90%) of which were reported as treatment related (Table 3). Ten (24%) patients experienced a serious AE; and 33 (79%) patients had a grade 3 or 4 TEAE (Table 3). AEs led to dose reduction or dose interruption in 48% and 38% of patients, respectively.

**Table 3:** Summary of treatment-emergent adverse events occurring in ≥10% of patients in the dose-confirmation cohort (phase 2; safety analysis set)

Event	TEAEs, *n* (%)			
	All grades	Grades 3 or 4^a^	Treatment related^b^	Treatment related, grades 3 or 4
Patients with any TEAE	39 (93)	33 (79)	38 (90)	30 (71)
Patients with any SAE	10 (24)	7 (17)	6 (14)	4 (10)
Blood and lymphatic system disorders	35 (83)	30 (71)	34 (81)	30 (71)
Neutropenia	34 (81)	29 (69)	34 (81)	29 (69)
Leukopenia	20 (48)	12 (29)	20 (48)	12 (29)
Anaemia	11 (26)	2 (5)	5 (12)	—
Gastrointestinal disorders	20 (48)	6 (14)	19 (45)	2 (4.8)
Nausea	13 (31)	2 (5)	12 (29)	1 (2.4)
Diarrhoea	9 (21)	0	8 (19)	—
Vomiting	7 (17)	0	7 (17)	—
Stomatitis	8 (19)	1 (2)	6 (14)	1 (2.4)
Abdominal pain	7 (17)	1 (2)	4 (10)	—
General disorders and administration-site conditions	16 (38)	2 (5)	12 (29)	2 (4.8)
Pyrexia	7 (14)	0	6 (14)	—
Fatigue	6 (14)	2 (5)	5 (12)	2 (4.8)
Asthenia	5 (12)	0	2 (5)	—
Investigations	15 (36)	2 (5)	8 (19)	1 (2.4)
Alanine aminotransferase level increased	6 (14)	1 (2)	2 (5)	1 (2.4)
Blood lactate dehydrogenase level increased	6 (14)	0	2 (5)	—
Musculoskeletal and connective tissue disorders	11 (26)	2 (5)	2 (5)	1 (2.4)^c^
Back pain	5 (12)	1 (2)	0	—
Nervous system disorders	17 (40)	6 (14)	14 (33)	4 (9.5)
Lethargy	8 (19)	2 (5)	8 (19)	2 (4.8)
Peripheral sensory neuropathy	6 (14)	2 (5)	6 (14)	2 (4.8)
Skin and subcutaneous tissue disorders	18 (43)	1 (2)	16 (38)	—
Alopecia	15 (36)	0	15 (36)	—
Palmar-plantar erythrodysaesthesia syndrome	11 (26)	1 (2)	7 (17)	—

*TEAE* treatment-emergent adverse event

^a^14 (33%) experienced grade 3 TEAEs; 18 (43%) experienced grade 4 TEAEs

^b^Includes TEAEs reported to be possibly or probably related to study drug, in the opinion of the investigator, or TEAEs with missing causality

^c^1 patient experienced treatment-related arthralgia

Neutropenia (*n* = 29, 69%) and leukopenia (*n* = 12, 29%) were the most common grade 3 or 4 TEAEs (Table 3), but febrile neutropenia of grade 3 or 4 was reported in only 2 (5%) patients. Two (5%) patients had TEAEs that led to treatment discontinuation: 1 patient had deep vein thrombosis (grade 4) and pulmonary embolism (grade 5; both considered “possibly related” to the study drug by the investigator), and the other patient developed peripheral sensory neuropathy (grade 3, considered “probably related” to the study drug).

There were 3 treatment-emergent deaths in this part of the study due to PD (*n* = 2) and pulmonary embolism (*n* = 1). QTcF interval prolongation was observed in 3 patients (grade 2 [*n* = 1], grade 3 [*n* = 2]), each of whom had at least 1 post-baseline value >480 ms.

## Discussion

In this study, the combination of eribulin mesilate administered at the approved single-agent full dose and schedule,^9,10^ with capecitabine given at a dose widely used as a single agent, was well tolerated and active with an ORR of 43%, which is considerably higher than the ORR of 9–12% observed for single-agent eribulin therapy in previous studies in patients with heavily pretreated MBC.^9,10,18,19^ Similarly, in patients with MBC, capecitabine has been associated with ORRs of 12–26% in later-line settings^10,13,20^ and 30% as first-line treatment.^21^ The ORR of eribulin in combination with capecitabine in the present study suggests additive and potentially synergistic activity of the combination. The efficacy of this combination is further supported by a high CBR (CR, PR, and SD ≥6 months, 57%), as well as prolonged median PFS and duration of response observed in this study. These benefits appeared to be maintained in patients with HER2-negative and triple-negative disease. The efficacy and tolerability of the combination may reflect both agents being given at, or close to, their full single-agent dosages. In addition, we can hypothesise that the vascular remodelling that is associated with eribulin^7^ may enhance the distribution and intratumoural penetration of capecitabine and its metabolites.

Toxicities seen with the combination were consistent with the known side effects of eribulin and capecitabine as monotherapy. Although the incidence of TEAEs was higher than with either drug given as a single agent,^9,10,22^ no new safety signal emerged with the combination. The most frequently reported AEs were typically managed by dose delays and reductions. Neutropenia was generally asymptomatic, with only 2 patients experiencing febrile neutropenia and 1 patient experiencing neutropenic sepsis in the phase 2 part of the study. Peripheral neuropathy was common, but only 2 (5%) patients experienced neuropathy greater than grade 2 in phase 2. In this phase of the study, although 79% of patients had a grade 3 or 4 TEAE, and 24% had events categorised as serious, only 2 (5%) patients discontinued treatment due to a TEAE.

Eribulin pharmacokinetics were comparable with published data and consistent across dose levels, schedules, and treatment cycles. Eribulin pharmacokinetics were unaffected by the coadministration of capecitabine, indicating that there were no drug–drug interactions. Likewise, there was no correlation between eribulin concentration and change in the QTcF in the dose-escalation phase of the study. In total, 5 patients in this study developed QT prolongation (phase 1b, *n* = 2; phase 2, *n* = 3). Based on independent cardiologist review of data from patients with QTc prolongation, all events were considered to be caused by other factors (e.g. hypokalaemia or pre-existing heart disease with ECG abnormalities) or were more likely related to other factors (i.e. the time course is not suggestive of a causal relationship). Therefore, these QTc data do not represent an emerging safety signal.

The tolerability, treatment duration, and delivered dose intensity of the eribulin and capecitabine combination compare favourably with those reported in the phase 3 study of docetaxel plus capecitabine, with remarkably similar efficacy.^12^ The addition of capecitabine to docetaxel improved OS and achieved an ORR of 42%; median time to progression was 6.1 months (compared to an ORR of 43% and median PFS of 7.2 months in the current study). In terms of toxicity, in the current study there were fewer grade 3 or 4 treatment-related TEAEs with the combination of eribulin and capecitabine than were seen with the combination of docetaxel and capecitabine (71% vs 96%, respectively) and less febrile neutropenia (5% [grade 3 or 4] vs 3% [grade 3] and 13% [grade 4], respectively.^12^ Treatment duration was also longer with the eribulin and capecitabine combination than with docetaxel and capecitabine combination (median, 5.6 vs 3.8 months, respectively), and fewer patients required dose reductions due to AEs (48% vs 65%, respectively).^12^ Therefore, we hypothesise that the combination of eribulin and capecitabine may offer similar efficacy with lower toxicity than docetaxel combined with capecitabine.

The limitations of the current study are its size, absence of randomisation, and lack of quality-of-life (QOL) data. Of note, the combination given as in the present study but with a different capecitabine regimen was recently evaluated in another single-arm, phase 2 feasibility study as adjuvant treatment in 77 patients with HER2-negative, oestrogen receptor-positive early-stage breast cancer. In that study, again the combination was feasible and the reported AEs were consistent with the known toxicities of each drug used alone.^23^ The lack of QOL data from the current study reflects it being a single-arm study. Interestingly, although the addition of capecitabine to docetaxel increased the incidence of gastrointestinal AEs and hand–foot syndrome, this did not result in worse QOL,^12^ presumably because the combination had greater anticancer efficacy. Assessment of QOL will be an important element of future trials of eribulin combined with capecitabine.

In conclusion, eribulin and capecitabine in combination showed promising antitumour activity in patients with MBC, with manageable tolerability. The combination demonstrated an ORR considerably higher than the ORR observed for single-agent eribulin therapy or capecitabine in previous studies of patients with MBC. Despite being one of the few regimens to significantly improve OS in patients with MBC, the combination of docetaxel and capecitabine is less widely used owing to toxicity concerns. The current study suggests that eribulin in combination with capecitabine may be an attractive option for patients in whom combination therapy is appropriate, such as those with rapidly progressing visceral disease. Further evaluation of the combination is warranted to establish whether it prolongs survival, without sacrificing QOL, compared to sequential single-agent chemotherapy for patients with MBC.

## Supplementary information

IRB List

Online Fig. S1

Online Fig. S2

Supplementary Resource

## Availability of data and material

The data in this publication are commercially confidential. Data requests should be made to the Corresponding Author.
