# Supplementary material for: A phase 1b/2, open-label, dose-escalation, and dose-confirmation study of eribulin mesilate in combination with capecitabine
Source: Br J Cancer. 2019 Feb 20;120(6):579–86. doi: 10.1038/s41416-018-0366-5 (PMC6461928; doi:10.1038/s41416-018-0366-5)
Supplement: Supplementary file 1 — IRB List [file 41416_2018_366_MOESM1_ESM.docx]

The list of IRBs and IECs is provided below.

| **Country** | **Site**  **Number** | **Principal**  **Investigator** | **Central and/or Local IRB/IEC Information** |
| --- | --- | --- | --- |
| United  Kingdom | 1001 | Prof. Evans | West of Scotland REC 1  Ground Floor, Tennent Institute  38 Church Street  Western Infirmary  Glasgow, G11 6NT |
| United  Kingdom | 1002 | Prof. Plummer | West of Scotland REC 1  Ground Floor, Tennent Institute  38 Church Street  Western Infirmary  Glasgow, G11 6NT |
| United  Kingdom | 1003 | Prof. Twelves | West of Scotland REC 1  Ground Floor, Tennent Institute  38 Church Street  Western Infirmary  Glasgow, G11 6NT |
| Russia | 2001 | Prof. Semiglazov | ***Central Ethics Committee:***  Ministry of Healthcare of the Russian Federation,  Ethics Council, 3 Rakhmanovsky Per.  Moscow, 127051  ***Local Ethics Committee:***  Local Ethics Committee within Federal State  Budgetary Institution: “Research Institute of  Oncology n.a. N.N. Petrov” under the Ministry of  Healthcare of the Russian Federation  68 Leningradskaya Str.  Pesochny-2  St. Petersburg, 197758 |
| Russia | 2002 | Dr. Manikhas | ***Central Ethics Committee:***  Ministry of Healthcare of the Russian Federation,  Ethics Council,  3 Rakhmanovsky Pereulok,  Moscow, 127051  ***Local Ethics Committee:***  Ethics Committee within St. Petersburg State  Budgetary Medical Institution: City Clinical  Oncology Center  56, Pr. Veteranov,  St. Petersburg, 198255 |
| Russia | 2004 | Dr. Fadeyeva | ***Central Ethics Committee:***  Ministry of Healthcare of the Russian Federation,  Ethics Council,  3 Rakhmanovsky Pereulok,  Moscow, 127051  ***Local Ethics Committee:***  Ethics Committee within State Budgetary  Healthcare Institution: Chelyabinsk Regional  Clinical Oncology Center  42 Blyukhera Ul.  Chelyabinsk, 454087 |
| Russia | 2005 | Dr. Roman | ***Central Ethics Committee:***  Ministry of Healthcare of the Russian Federation,  Ethics Council, 3 Rakhmanovsky Per.  Moscow, 127051  ***Local Ethics Committee:***  Local Ethics Committee within State Budgetary  Healthcare Institution: Leningrad Regional  Oncology Center  37/39 Liteynyi pr.,  St. Petersburg, 191104 |
| Russia | 2007 | Dr. Udovitsa | ***Central Ethics Committee:***  Ministry of Healthcare of the Russian Federation,  Ethics Council,  3 Rakhmanovsky Pereulok,  Moscow, 127051  ***Local Ethics Committee:***  The Independent Ethics Committee within “Arte  Med Assistants” LLC.  22-24 Nevsky Pr.,  St. Petersburg, 191186 |
| Russia | 2008 | Dr. Vladimirov | ***Central Ethics Committee:***  Ministry of Healthcare of the Russian Federation,  Ethics Council,  3 Rakhmanovsky Pereulok,  Moscow, 127051  ***Local Ethics Committee:***  Local Ethics Committee within State Budgetary  Medical Institution of Stavoropol Region:  Pyatigorsk Oncology Dispensary  31 Kalinina Pr.,  Pyatigorsk, Stavropol Region, 357502 |
| Bulgaria | 3001 | Dr. Ivanova | Ethics Committee for Multicenter Trials  5 “Sveta Nedelya” Sq.  1000 Sofia |
| Bulgaria | 3002 | Dr. Koynova | Ethics Committee for Multicenter Trials  5 “Sveta Nedelya” Sq.  1000 Sofia |
| Bulgaria | 3003 | Assoc. Prof.  Timcheva | Ethics Committee for Multicenter Trials  5 “Sveta Nedelya” Sq.  1000 Sofia |
| Bulgaria | 3004 | Dr. Tomova | Ethics Committee for Multicenter Trials  5 “Sveta Nedelya” Sq.  1000 Sofia |
