# Supplementary figures and images for: A phase 1b/2, open-label, dose-escalation, and dose-confirmation study of eribulin mesilate in combination with capecitabine
Source: Br J Cancer. 2019 Feb 20;120(6):579–86. doi: 10.1038/s41416-018-0366-5 (PMC6461928; doi:10.1038/s41416-018-0366-5)

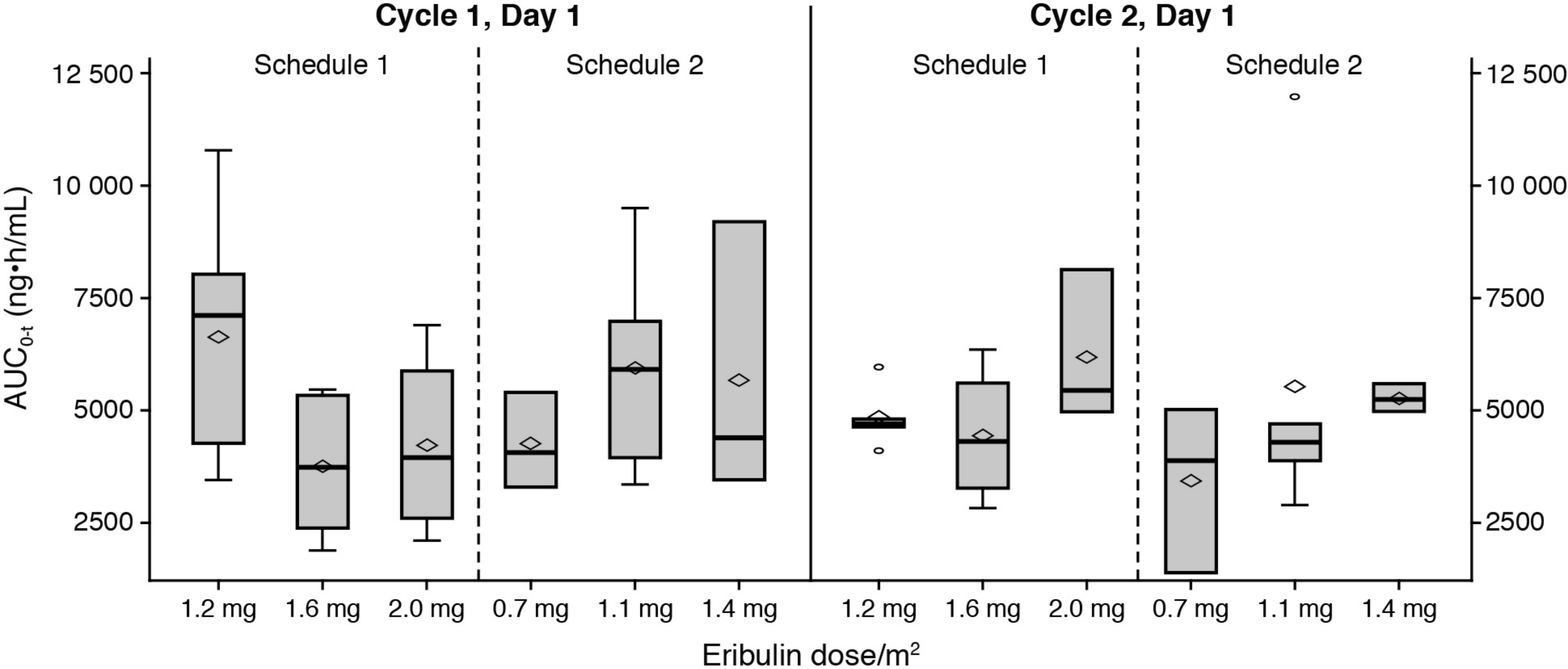

$AUC_{0-t}$ , area under the concentration–time curve from time 0 to last measurable concentration

Supplement: Supplementary file 2 — Online Fig. S1 [file 41416_2018_366_MOESM2_ESM.pdf]

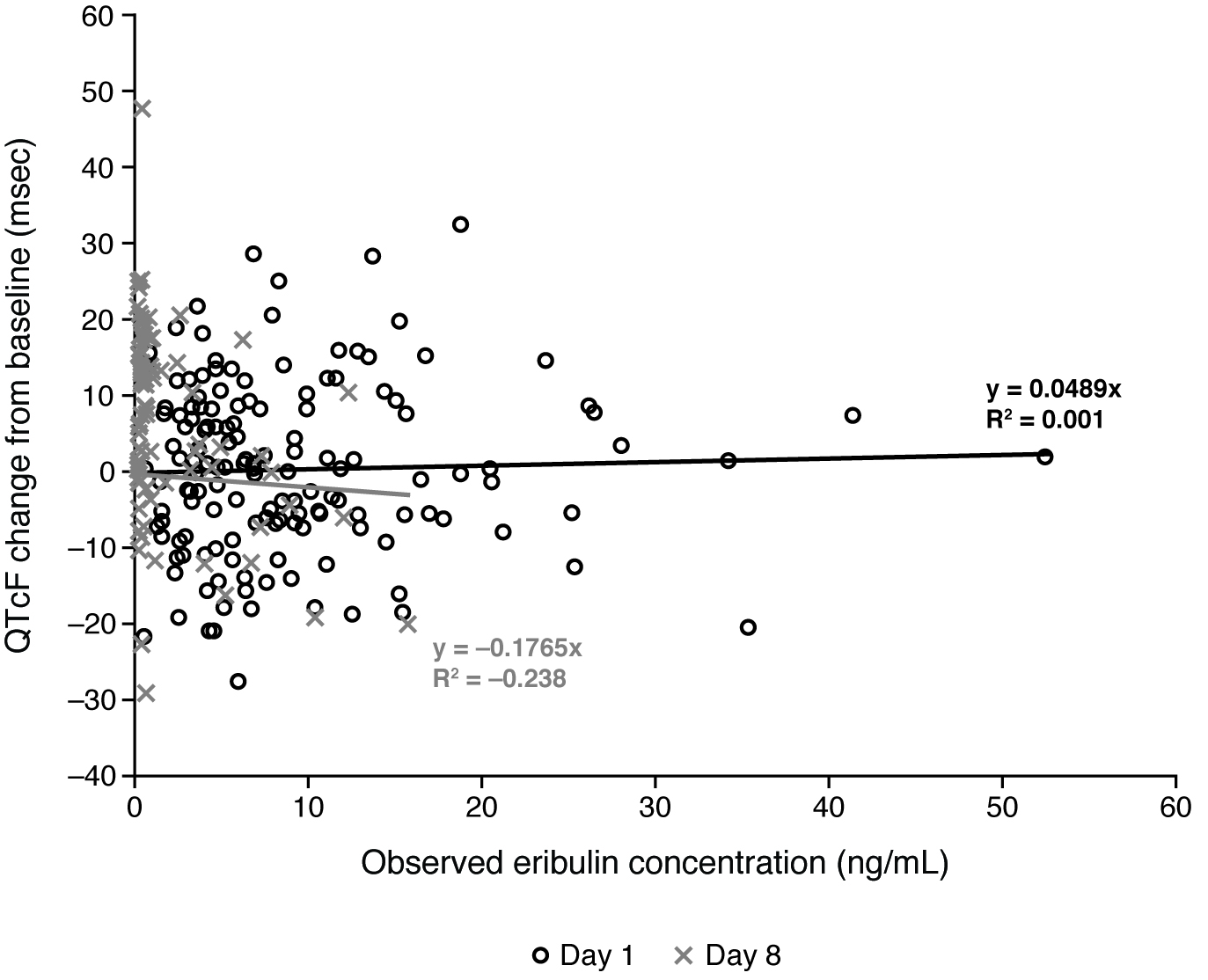

Supplement: Supplementary file 3 — Online Fig. S2 [file 41416_2018_366_MOESM3_ESM.jpg]
