# Supplementary material for: A phase 1b/2, open-label, dose-escalation, and dose-confirmation study of eribulin mesilate in combination with capecitabine
Source: Br J Cancer. 2019 Feb 20;120(6):579–86. doi: 10.1038/s41416-018-0366-5 (PMC6461928; doi:10.1038/s41416-018-0366-5)
Supplement: Supplementary file 4 — Supplementary Resource [file 41416_2018_366_MOESM4_ESM.docx]

**SUPPLEMENTARY RESOURCE**

**Supplementary Fig. S1** Exposure to capecitabine on day 1 of cycles 1 and 2 in the dose-escalation cohort (phase 1b, both treatment schedules included); capecitabine was dosed at 1000 mg/m^2^ twice daily on days 1–14 of 21-day cycles

**Supplementary Fig. S2** Relationship between plasma concentration of eribulin and change in QTcF from baseline in the dose-escalation cohort (phase 1b); black and grey lines represent linear approximation curves for day 1 and day 8, respectively

QTcF, QT corrected for heart rate using Fridericia's method.

**Supplementary Tables**

**Supplementary Table S1** Selected additional eligibility criteria for study enrolment.

| **Study phase** | **Additional inclusion criteria** |
| --- | --- |
| 1b | - Resistant/refractory to approved therapies (defined as progressive disease during or within 6 months after the last anticancer therapy) or for whom single-agent capecitabine at this dose level and schedule were a reasonable treatment option in the opinion of the investigator |
|  | - For patients who had received capecitabine, all of its related toxicities must have been completely resolved |
|  | - An ECOG PS of ≤ 2 |
|  | - Males who were not abstinent or had not undergone a successful vasectomy, who were partners of women of childbearing potential, must have used, or their partners must have used, a highly effective method of contraception (e.g., condom + spermicide, condom + diaphragm with spermicide, IUD) for at least 1 menstrual cycle prior to starting study drug and throughout the entire study period, and for 30 days (longer, if appropriate) after the last dose of study drug. Those with partners using hormonal contraceptives must also have been using an additional approved method of contraception (as described previously) |
| 1b and 2 | - Adequate bone marrow function:   - Absolute neutrophil count ≥1.5 x 10^9^/L   - Haemoglobin ≥10.0 g/dL   - Platelet count ≥100 x 10^9^/L |
|  | - Adequate liver function:   - Bilirubin ≤1.5 times the upper limit of normal   - Alkaline phosphatase, alanine aminotransferase, and aspartate aminotransferase ≤3 times the upper limit of normal (≤5 times the upper limit of normal for patients with liver metastases) |
|  | - Adequate renal function:   - Creatinine clearance ≥50 mL/min as per the Cockcroft–Gault formula or radioisotope measurement |
|  | - Life expectancy of >3 months |
|  | - Females of childbearing potential must have had a negative urine or serum ßhCG result at visit 1 (screening) and prior to starting study drugs on day 1. Women of childbearing potential must have agreed to be abstinent or to use highly effective methods of contraception (e.g., condom + spermicide, condom + diaphragm with spermicide, IUD, or have a vasectomised partner) for at least 1 menstrual cycle prior to starting study drugs and throughout the entire study period, and for 30 days (longer, if appropriate) after the last dose of study drugs. Women using hormonal contraceptives must also have been using an additional approved method of contraception (as described previously). Perimenopausal women must have been amenorrhoeic for at least 12 months to be considered of nonchildbearing potential |
|  | **Additional exclusion criteria** |
| 1b and 2 | - Significant cardiovascular impairment (history of congestive heart failure >NYHA grade II, unstable angina or myocardial infarction within the past 6 months, or serious cardiac arrhythmia) |
|  | - Electrocardiogram with QTc interval >470 msec (as measured either by Bazett’s or Fridericia’s formula) |

ßhCG, beta human chorionic gonadotropin; ECOG, Eastern Cooperative Oncology Group performance status; IUD, intrauterine device; NYHA, New York Heart Association.

**Supplementary Table S2** Criteria for dose modifications during study treatment for (a) eribulin mesilate and (b) capecitabine

(a)

| **Schedule** | **Circumstance** | **Dose adjustment** | | | |
| --- | --- | --- | --- | --- | --- |
| 1 | - ANC <1.0 x 10^9^/L, *or* - Platelets <75 x 10^9^/L, *or* - Toxicity from a previous cycle >grade 2 | Delay administration until these parameters have recovered to the required criteria | | | |
|  | - Haematologic toxicity - Grade 4 neutropenia >7 days, *or* - Grade 3 or 4 neutropenia complicated by fever and/or infection | **Starting dose** | **First event** | **Second event** | **Third event** |
|  |  | 1.2 mg/m^2^ | Discontinue |  |  |
|  |  | 1.6 mg/m^2^ | Reduce to 1.2 mg/m^2^ | Discontinue |  |
|  |  | 2.0 mg/m^2^ | Reduce to 1.6 mg/m^2^ | Reduce to 1.2 mg/m^2^ | Discontinue |
|  | Treatment delayed for >2 weeks | Reduce dose to the next lower dose level or discontinue (per table above) | | | |
| 2 | - ANC <1.0 x 10^9^/L *or* - Platelets <75 x 10^9^/L, *or* - Non-haematologic toxicity >grade 2 (except inadequately treated nausea and/or vomiting) | Delay administration until these parameters have recovered to the required criteria | | | |
|  | - Hematologic toxicity - Grade 4 neutropenia >7 days, *or* - Grade 3 or 4 neutropenia complicated by fever and/or infection | **Starting dose** | **First event** | **Second event** | **Third event** |
|  |  | 0.7 mg/m^2^ | Discontinue |  |  |
|  |  | 1.1 mg/m^2^ | Reduce to 0.7 mg/m^2^ | Discontinue |  |
|  |  | 1.4 mg/m^2^ | Reduce to 1.1 mg/m^2^ | Reduce to 0.7 mg/m^2^ | Discontinue |
|  | - Treatment omitted on day 8 | Resume treatment as scheduled on day 1 of the next cycle. However, the dose of eribulin mesilate for the next and any subsequent cycles is reduced to the next lower dose level or discontinued according to the above table | | | |

(b)

| **Circumstance** | **Dose adjustment** | | | | |
| --- | --- | --- | --- | --- | --- |
| Toxicity requiring dose reduction during the first 14 days of a treatment cycle | Reduce dose appropriately in subsequent cycles | | | | |
| Toxicity between scheduled visits | Patient is encouraged to contact the clinic immediately for further instructions or treatment | | | | |
| **Hand/foot/skin reaction** | **Grade^a^** | **First event/dose** | **Second event/dose** | **Third event/dose** | **Fourth event/dose** |
| Numbness, dysesthesia/ paresthesia, tingling, painless swelling or erythema of the hands and/or feet and/or discomfort that does not disrupt normal activities | 1 | Maintain dose level (1000 mg/m^2^) twice daily | | | |
| Painful erythema and swelling of the hands and/or feet and/or discomfort affecting the patient’s activities of daily living | 2 | Delay^b^ then  1000 mg/m^2^ twice daily | Delay^b^ then  750 mg/m^2^ twice daily | Delay^b^ then  500 mg/m^2^ twice daily | Discontinue |
| Moist desquamation, ulceration, blistering or severe pain of the hands and/or feet and/or severe discomfort that cause the patient to be unable to work or perform activities of daily living | 3 | Delay^b^ then  750 mg/m^2^ twice daily | Delay^b^ then  500 mg/m^2^ twice daily | Discontinue | Discontinue |

^a^Grade according to capecitabine product monograph (Reference: Xeloda product monograph. Hoffmann-La Roche Limited. www.rochecanada.com/content/dam/roche_canada/en_CA/documents/Research/ClinicalTrialsForms/Products/ConsumerInformation/MonographsandPublicAdvisories/Xeloda/Xeloda_PM_E.pdf. Accessed March 14, 2016).
^b^Stop treatment immediately and delay until resolved to grade 0 to 1.
ANC, absolute neutrophil count.

**Supplementary Table S3** Additional information on study assessments

| **Assessment of tumour response** |
| --- |
| - Based on RECIST version 1.1 - Computed tomography scans of chest, abdomen, pelvis, and other areas as necessary - Tumour assessments were to be performed within 28 days prior to the start of study treatment and every 6 weeks from the first dose of study drugs, or as clinically indicated to confirm response or disease progression |
| **Safety assessment** |
| - Safety was assessed by monitoring and recording adverse events (AEs) and serious AEs, clinical laboratory parameters, vital signs, and electrocardiograms |

AUC_0-∞_, area under the concentration–time curve from zero (pre-dose) extrapolated to infinity; CL, systemic clearance; C_max_, maximum plasma concentration; RECIST, Response Evaluation Criteria in Solid Tumors.

**Supplementary Table S4** Baseline patient and disease characteristics for the dose-escalation (phase 1b) cohort

| **Characteristic** | **Phase 1** | |
| --- | --- | --- |
|  | **Schedule 1 (n = 19)** | **Schedule 2 (n = 15)** |
| Median age, years (range) | 62 (40–79) | 61 (38–69) |
| Gender, n (%) |  |  |
| Male | 10 (53) | 5 (33) |
| Female | 9 (47) | 10 (67) |
| Race, n (%) |  |  |
| White | 19 (100) | 15 (100) |
| ECOG PS, n (%) |  |  |
| 0 | 8 (42) | 5 (33) |
| 1 | 11 (58) | 8 (53) |
| 2 | 0 | 2 (13) |
| Prior anticancer therapy, n (%) |  |  |
| 1 | 1 (5) | 5 (33) |
| 2 | 4 (21) | 4 (27) |
| 3 | 8 (42) | 5 (33) |
| >3 | 6 (32) | 1 (7) |
| Primary tumour location, *n* (%) |  |  |
| Large intestine | 6 (32) | 1 (7) |
| Lung and bronchus | 2 (11) | 2 (13) |
| Breast | 2 (11) | 3 (20) |
| Rectum | 3 (16) | 0 |
| Ovary | 1 (5) | 1 (17) |
| Other^a^ | 4 (21) | 8 (53) |
| Unknown | 1 (5) | 0 |

^a^Other locations of primary tumour in schedule 1: pleura (n = 2), vulva (n = 1), gallbladder and extrahepatic bile ducts (n = 1). Other locations of primary tumour in schedule 2: connective and soft tissue (n = 1), oesophagus (n = 1), kidney (n = 1), liver (n = 1), pancreas (n = 1), prostate gland (n = 1), urinary bladder (n = 1), uterus (n = 1).

ECOG PS, Eastern Cooperative Oncology Group Performance Status.

**Supplementary Table S5** Pharmacokinetic parameters for eribulin on day 1 of cycles 1 and 2 of each treatment schedule in the dose-escalation (phase 1b) part of the study

| **Parameter** | **Schedule 1** | | | **Schedule 2** | | |
| --- | --- | --- | --- | --- | --- | --- |
|  | **Eribulin mesilate 1.2 mg/m^2^ + capecitabine  1000 mg/m^2^** | **Eribulin mesilate 1.6 mg/m^2^ + capecitabine  1000 mg/m^2^** | **Eribulin mesilate 2.0 mg/m^2^ + capecitabine  1000 mg/m^2^** | **Eribulin mesilate 0.7 mg/m^2^ + capecitabine 1000 mg/m^2^** | **Eribulin mesilate 1.1 mg/m^2^ + capecitabine  1000 mg/m^2^** | **Eribulin mesilate 1.4 mg/m^2^ + capecitabine  1000 mg/m^2^** |
| Cycle 1 | (n = 8) | (n = 6) | (n = 5) | (n = 3) | (n = 5) | (n = 5) |
| C_max_ (ng/mL) | 357 (108) | 1070 (1570) | 636 (117) | 218 (72) | 319 (101) | 505 (110) |
| AUC_0-∞_ (ng·h/mL) | 606 (288) | 1480 (979) | 1130 (534) | 422 (48) | 507 (148) | 785 (258) |
| t_1/2_ (h) | 36.2 (12) | 47.4 (15) | 39.6 (3) | 26.6 (2) | 35.9 (6) | 33.5 (6) |
| CL (l/h) | 6.2 (3)^a^ | 2.9 (1) | 3.9 (2) | 3.6 (0.1) | 4.6 (1) | 3.2 (1) |
| Cycle 2 | (n = 5) | (n = 5) | (n = 5) | (n = 3) | (n = 4) | (n = 4) |
| C_max_ (ng/mL) | 498 (55) | 1080 (1430) | 493 (279) | 1140 (1590) | 337 (94) | 359 (240) |
| AUC_0-∞_ (ng·h/mL) | 578 (449) | 1530 (1120) | 1020 | 517 | 425 (73) | 686 (110) |
| t_1/2_ (h) | 29.2 (5) | 41.3 (12) | 43.3 | 28.8 | 34.9 (6) | 39.6 (8) |
| CL (L/h) | 5.9 (3) | 4.1 (2) | 3.9 | 2.6 | 5.2 (0.4) | 3.3 (1) |

All values are presented as mean (SD).

AUC_0-∞_, area under the concentration−time curve from zero (pre-dose) extrapolated to infinity; CL, systemic clearance; C_max_, maximum plasma concentration; SD, standard deviation; t_1/2_, terminal elimination half-life

**Supplementary Table S6** Pharmacokinetic parameters for capecitabine and 5-fluorouracil on day 1 of cycles 1 and 2 (schedule 1 and schedule 2) in the dose-escalation part of the study (phase 1b)

| **Parameter** | **Eribulin mesilate**  **1.2 mg/m^2^ +**  **capecitabine**  **1000 mg/m^2^** | | **Eribulin mesilate  1.6 mg/m^2^ +**  **capecitabine**  **1000 mg/m^2^** | | **Eribulin mesilate**  **2.0 mg/m^2^ +**  **capecitabine 1000 mg/m^2^** | | **Eribulin mesilate**  **0.7 mg/m^2^ +**  **capecitabine**  **1000 mg/m^2^** | | **Eribulin mesilate**  **1.1 mg/m^2^ +**  **capecitabine 1000 mg/m^2^** | | **Eribulin mesilate**  **1.4 mg/m^2^ +**  **capecitabine 1000 mg/m^2^** | | **Literature**  **value^b^** |
| --- | --- | --- | --- | --- | --- | --- | --- | --- | --- | --- | --- | --- | --- |
|  | **Cycle 1**  **(n = 8)** | **Cycle 2**  **(n = 5)** | **Cycle 1**  **(n = 5)** | **Cycle 2**  **(n = 4)** | **Cycle 1**  **(n = 4)** | **Cycle 2**  **(n = 3)** | **Cycle 1**  **(n = 3)** | **Cycle 2**  **(n = 3)** | **Cycle 1**  **(n = 6)** | **Cycle 2**  **(n = 5)** | **Cycle 1 (n = 3)** | **Cycle 2 (n = 3)** |  |
| Capecitabine |  |  |  |  |  |  |  |  |  |  |  |  |  |
| AUC_0-t_ (ng·h/mL) | 6650 (2530) | 4860 (683) | 3780  (1650) | 4460  (1520) | 4260  (2090) | 6210  (1700) | 4270  (1060) | 3440  (1850) | 5960  (2220) | 5560  (3660) | 5700 (3080) | 5290 (306) | ― |
| C_max_ (ng/mL) | 7310 (4620) | 4280 (1030) | 3350  (1090) | 2550  (1370) | 4280  (3000) | 7200  (3810) | 5260  (1660) | 3200  (2250) | 8200  (4670) | 6740  (5840) | 3430 (1350) | 3480 (1330) | ― |
| t_max_ (h)^a^ | 1.1  (0.6–2.1) | 0.6  (0.5–3.0) | 1.0  (0.6–2.2) | 0.8  (0.5–3.0) | 0.7  (0.5–1.1) | 1.1  (0.7–2.2) | 1.1  (0.6–2.1) | 1.5  (1.0-2.3) | 0.9  (0.3­­–2.0) | 0.6  (0.3–2.1) | 1.0  (0.6–3.1) | 2.1  (0.8–3.1) | 1.5 |
| t_1/2_ (h) | 0.4 (0.1) | 0.3 (0.0) | 0.6 | 0.6 | 0.4 (0.0) | NC | 0.5 | 0.5 | 0.3 (0.1) | 0.5 (0.2) | 1.7 | 0.8 (0.5) | 0.8 |
| 5-fluorouracil |  |  |  |  |  |  |  |  |  |  |  |  |  |
| AUC_0-t_ (ng·h/mL) | 642  (565) | 836  (886) | 369  (108) | 391  (48.9) | 659  (139) | 751  (58.7) | 1290  (1280) | 1180  (1160) | 620  (103) | 513  (36.7) | 537  (262) | 193 (151) | ― |
| C_max_ (ng/mL) | 493  (463) | 563  (438) | 304  (97.8) | 218  (142) | 402  (112) | 625  (136) | 970  (872) | 588  (525) | 582  (246) | 410  (147) | 390  (256) | 162  (44) | ― |
| t_max_ (h)^a^ | 1.1  (0.7–4.1) | 1.1  (1.0–3.0) | 2.0  (0.6–2.2) | 2.0  (1.1–4.0) | 0.7  (0.5–1.1) | 1.1  (0.7–2.2) | 1.1  (1.1–2.1) | 2.3  (1.0–2.5) | 1.1  (0.6–2.0) | 1.1  (0.5–2.1) | 2.1  (0.6–3.1) | 3.0  (0.8–4.1) | 2.0 |
| t_1/2_ (h) | 0.6 | 0.7 | NC | NC | NC | NC | 0.7 | NC | NC | NC | NC | NC | 0.8 |

Values are presented as mean (SD) unless stated otherwise.

^a^Median (min–max)

AUC_0-t_, area under the concentration−time curve from time 0 to last measurable concentration; C_max_, maximum plasma concentration; SD, standard deviation; t_1/2_, terminal elimination half-life; t_max_, time to maximum plasma concentration.

^b^Reference: Xeloda (capecitabine) tablets [prescribing information]. San Francisco, CA: Genentech, Inc, 2015.

**Supplementary Table S7** Tumour response overall and in patients with HER2-negative or triple-negative disease in the dose-confirmation (phase 2) part of the study (investigator assessment; full analysis set)

| Response, n (%) | HER2 Negative | | HER2 positive  (n = 4) | HER2 unknown/ doubtful** (n = 5) | Overall (n = 42) |
| --- | --- | --- | --- | --- | --- |
|  | Triple negative (n = 16) | Non-triple negative* (n = 17) |  |  |  |
| Complete response | 0 | 0 | 0 | 1 (20) | 1 (2) |
| Partial response | 6 (38) | 9 (53) | 1 (25) | 1 (20) | 17 (41) |
| Stable disease | 5 (31) | 7 (41) | 1 (25) | 3 (60) | 16 (38) |
| Progressive disease | 3 (19) | 0 | 0 | 0 | 3 (7) |
| Not evaluable | 2 (13) | 1 (6) | 2 (50) | 0 | 5 (12) |
| Objective response rate  (95% CI) | 6 (38)  (15.2, 64.6) | 9 (53)  (27.8, 77.0) | 1 (25)  (0.6, 80.6) | 2 (40)  (5.3, 85.3) | 18 (43)  (27.7, 59.0) |
| Clinical benefit rate  (95% CI) | 9 (56)  (29.9, 80.2) | 12 (71)  (44.0, 89.7) | 1 (25)  (0.6, 80.6) | 2 (40)  (5.3, 85.3) | 24 (57)  (41.0, 72.3) |
| Disease control rate  (95% CI) | 11 (69)  (41.3, 89.0) | 16 (94)  (71.3, 99.9) | 2 (50)  (6.8, 93.2) | 5 (100)  (47.8, 100) | 34 (81)  (65.9, 91.4) |
|  |  |  |  |  |  |
| Median PFS (months)  (95% CI) | 4.5  (1.2, NE) | 7.2  (5.4, 9.8) | 5.4  (3.2, 7.7) | 13.7  (3.0, NE) | 7.2  (4.5, 10.8) |

Note: *Includes patients with HR positive (n = 16) and HR status unknown (n = 1). ** Her-2 status unknown/doubtful (2+ and FISH not done): ER negative and PR negative, n=2; ER positive and PR positive; n=1; ER not done, and PR not done, n=1. 

CI, confidence interval; HER2, human epidermal growth factor receptor 2; HR, hormone receptor; NE, not estimable, PFS, progression-free survival.

**Supplementary Table S8** Dose modifications by study drug in the dose-confirmation cohort (phase 2)

| **Parameter** | **Capecitabine (n = 42)** | **Eribulin mesilate (*n* = 42)** |
| --- | --- | --- |
| Cycles received, median (range) | 8 (1–46) | |
| Dose intensity (mg/m^2^/day) per patient, mean (SD) | 3204.1 (582.7) | 0.2 (0.3) |
| Received dose as percentage of planned dose per patient, median (range) | 100 (4–119) | 99 (50–102) |
| Dose reductions, n (%) | 10 (24) | 18 (43) |
| Dose omissions, n (%) | 3 (7) | 16 (38) |
| Dose delays, n (%) | 10 (24) | 23 (55) |

SD, standard deviation.
